# Spontaneous Spinal Epidural Hematoma Presenting as an Acute Stroke Mimic: A Diagnostic Challenge

**DOI:** 10.7759/cureus.90544

**Published:** 2025-08-20

**Authors:** Yen Yeong Poh, Ying Hao Christopher Seet

**Affiliations:** 1 Neurology, National Neuroscience Institute, Singapore, SGP

**Keywords:** acute hemiparesis, non-traumatic hematoma, spontaneous hemorrhage, spontaneous spinal epidural hematoma, stroke mimic

## Abstract

Given the limited time window (typically less than 4.5 hours from the last known well time) during which thrombolysis can be administered for acute ischemic stroke, accurate diagnosis and the efficient exclusion of contraindications and mimics are essential. One such mimic is that of spontaneous spinal epidural hematoma (SSEH), where diagnosis can be challenging, especially without an MRI. In this report, we describe a case of a patient who presented within the window for thrombolysis with a sudden onset of neck pain and right sensorimotor symptoms mimicking an acute cerebral infarction, who was found to have SSEH. High clinical suspicion with careful review of available neuroimaging (CT angiogram) led to prompt diagnosis and hence avoided administration of antithrombotic agents, which could have been detrimental.

## Introduction

Acute hemiparesis presenting within 4.5 hours of onset raised the possibility of acute ischemic stroke and the need to consider whether the patient is a candidate for intravenous (IV) thrombolysis [1]. Careful and prompt consideration for contraindications and mimics needs to be performed. Although some mimics, such as hyperglycemia or migraines, would likely not result in detrimental outcomes even if IV thrombolysis was given [2], mimics such as spontaneous spinal epidural hematoma (SSEH) have been shown to have worse outcomes when IV thrombolysis was inadvertently given [3]. We report a case of SSEH in a patient who presented with sudden onset right hemiparesis with neck pain, where high clinical suspicion and careful review of “code stroke” neuroimaging (CT angiogram from arch of aorta to circle of Willis) led to diagnosis and avoidance of inappropriate administration of antithrombotic agents.

## Case presentation

A 69-year-old man developed sudden right-sided weakness accompanied by neck pain approximately two hours after receiving a full-body massage. He was brought promptly to the emergency department within two hours of symptom onset and was triaged to priority level 1, reserved for critically ill patients requiring immediate attention.

His past medical history included atrial fibrillation with bradycardia, hypertension, and hyperlipidemia, for which he was on rivaroxaban and atorvastatin. On arrival, his vital signs were blood pressure 155/93 mmHg, temperature 37.1°C, heart rate 49 beats per minute, respiratory rate 20 breaths per minute, oxygen saturation 97%, and random capillary blood glucose 12.3 mmol/L.

Neurological examination revealed neck pain and stiffness, with Medical Research Council [4] grade 4 out of 5 weakness in the right upper and lower limbs and reduced right-sided sensation below the neck. Significant negative findings included the absence of facial weakness, normal pupillary responses, and normal plantar reflexes. Cranial nerve examination was otherwise normal. Laboratory tests showed a normal platelet count but a prolonged prothrombin time of 18.3 seconds, likely attributable to rivaroxaban.

**Figure 1:**
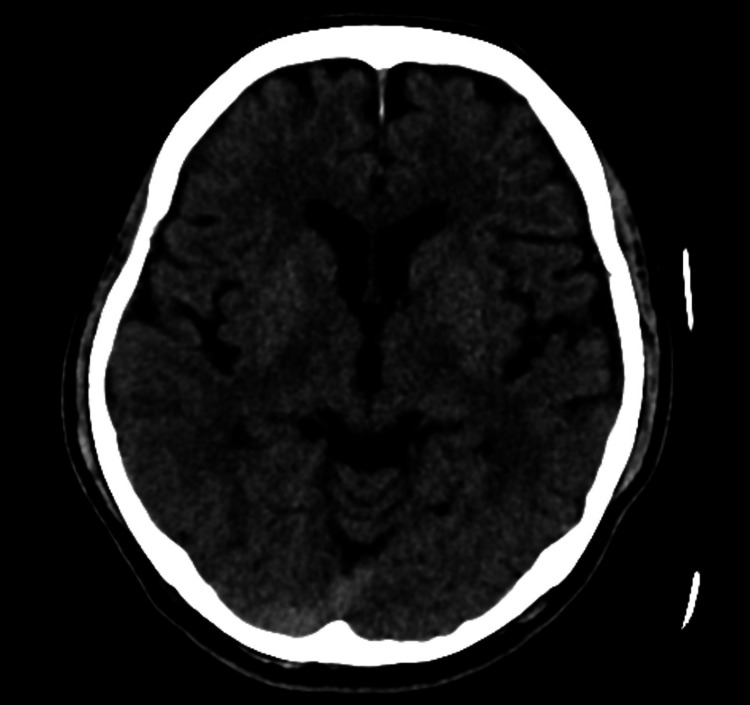
Plain CT brain revealed no acute intracranial hemorrhage, territorial infarct, or mass effect CT: computed tomography

**Figure 2:**
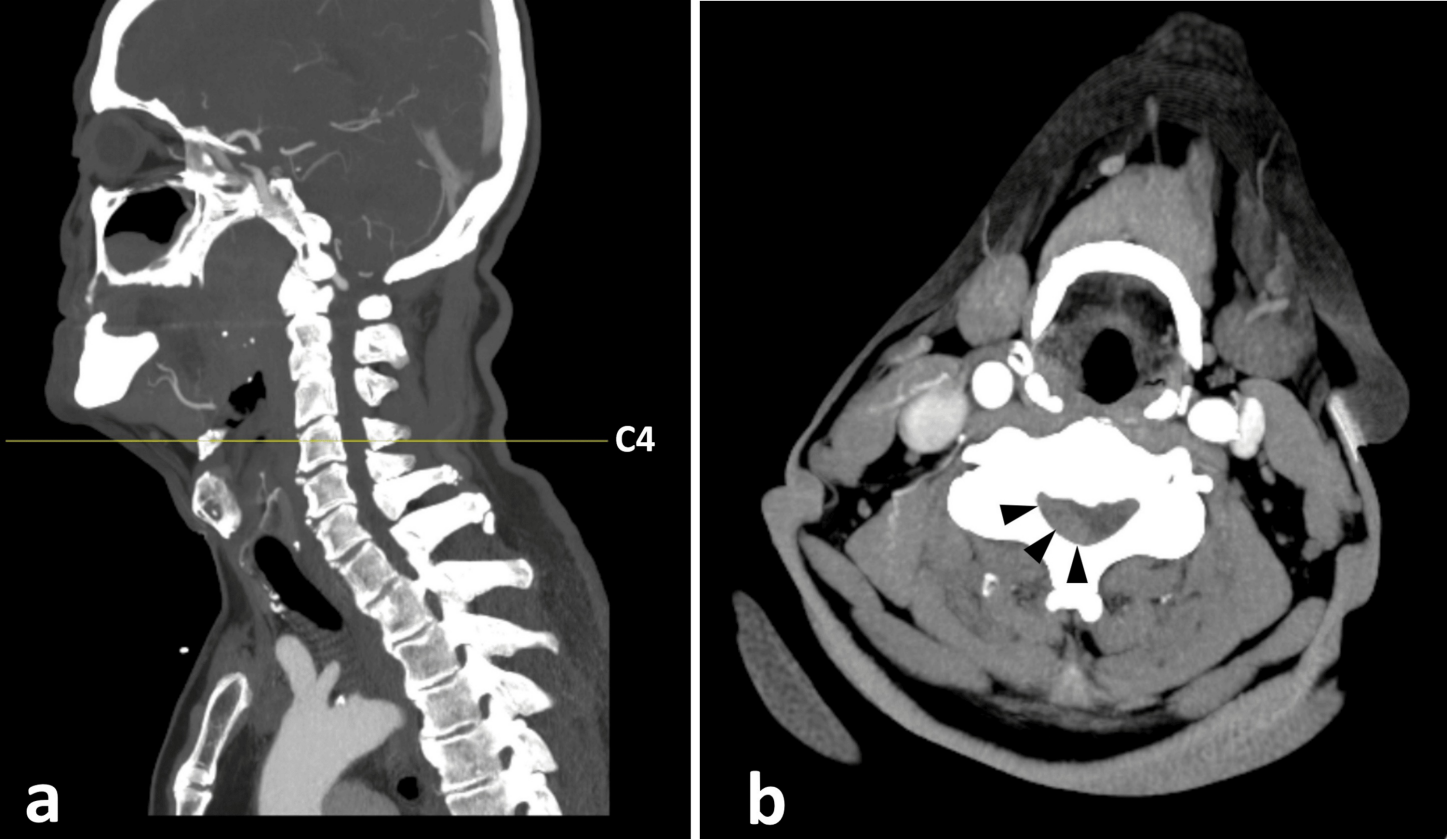
CT angiogram from the arch of the aorta to the circle of Willis showed a mildly hyperdense and mildly enhancing epidural lesion (black arrows) centered at C4 (extending from C3 to C5) with compression and contralateral displacement of the cervical cord suggestive of a mass lesion or an epidural hematoma. (a) Sagittal view with yellow line indicating cord level of image (b). (b) Axial view at the level indicated on image (a) CT: computed tomography

Given the acute onset of symptoms within two hours, a code stroke was activated. An urgent CT of the brain revealed no acute intracranial hemorrhage, territorial infarction, or mass effect (Figure 1). CT angiography from the aortic arch to the circle of Willis showed no intracranial or extracranial large vessel occlusion or critical stenosis. However, careful evaluation of the cervical spine on the same images demonstrated a mildly hyperdense enhancing epidural lesion centered at C4 and extending from C3 to C5 (Hounsfield unit 60). This lesion caused compression and contralateral displacement of the cervical cord, raising suspicion of a mass lesion or epidural hematoma (Figure 2).

**Figure 3:**
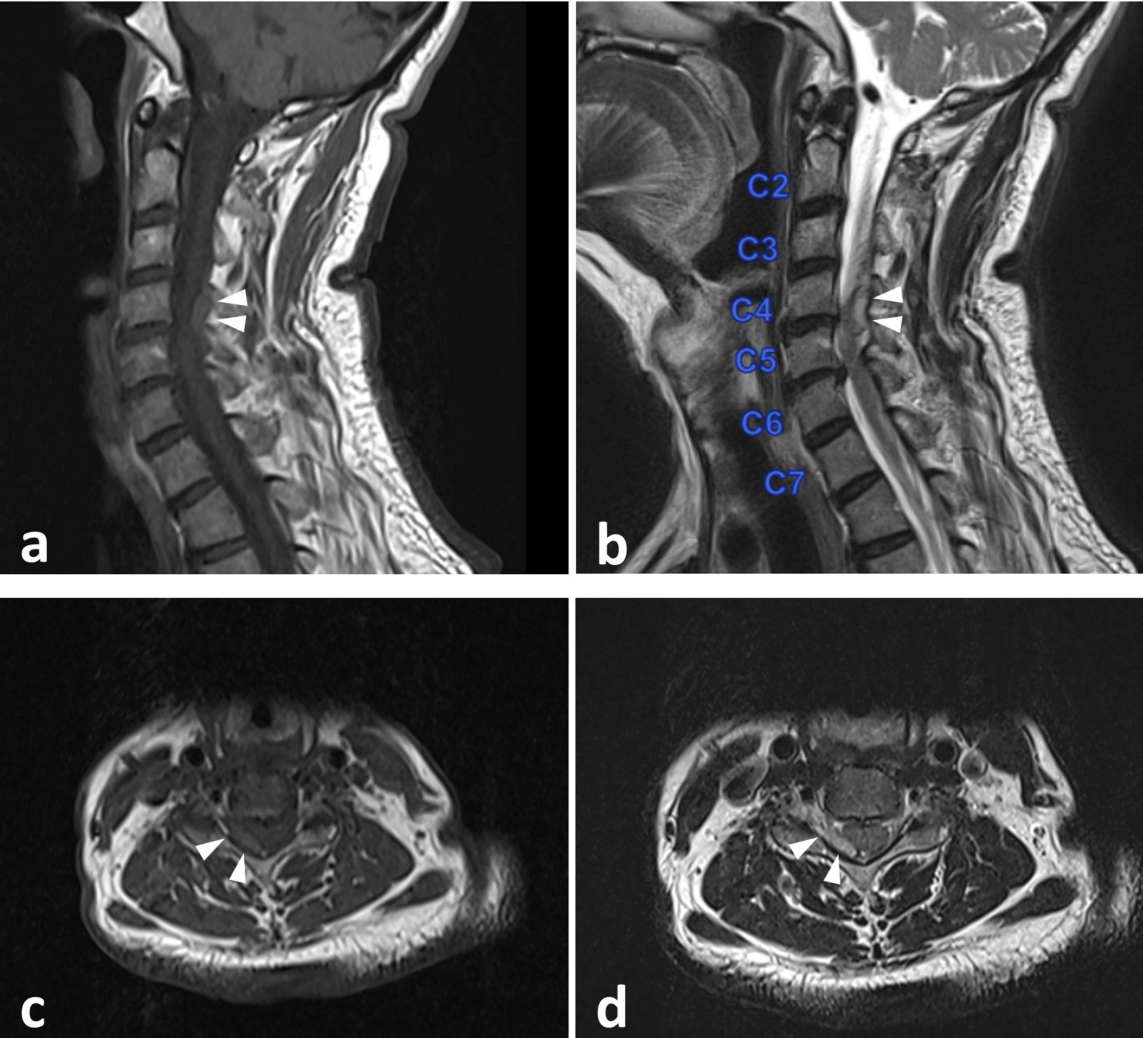
MRI of the cervical spine on day 2 showed a right posterolateral non-enhancing epidural collection spanning C3-4 to C5-6 levels, measuring 1.4 x 0.4 x 3.4 cm, which was hyperintense on the T1-weighted sequence, suggestive of hematoma (white arrows). (a) Sagittal T1-weighted sequence. (b) Sagittal T2-weighted sequence. (c) Axial T1-weighted sequence. (d) Axial T2-weighted sequence MRI: magnetic resonance imaging

Given these findings, and given that the patient was anticoagulated, IV thrombolysis was withheld, rivaroxaban was stopped, and the spine team was consulted. A subsequent MRI of the cervical spine confirmed a right posterolateral non-enhancing epidural collection spanning C3-4 to C5-6 levels, measuring 1.4 × 0.4 × 3.4 cm. The lesion appeared hyperintense on T1-weighted sequences, consistent with an epidural hematoma (Figure 3).

As the patient’s neurological deficits were mild and showed no progression over the next week, a conservative management strategy was adopted. Treatment included temporary cessation of rivaroxaban, bed rest, cervical immobilization with a collar, oral analgesia, and IV dexamethasone. On day 8 of admission, a repeat MRI of the cervical spine showed an interval reduction in the size of the hematoma (1.1 × 0.2 × 2.4 cm). His right-sided weakness and neck pain improved gradually, and he was discharged home on day 8 without significant disability. At the six-week follow-up, he remained asymptomatic with no residual neurological deficits.

## Discussion

Jackson first described SSEH in 1897 [5]. It is a rare spinal emergency with an estimated incidence of 0.1 per 100,000 patients per year [6]. While most patients present in their 60s or 70s, cases have been reported across all age groups, from 6 months to the late 80s [7]. This demographic coincides with that of patients with acute ischemic stroke. Although the exact pathophysiology of SSEH remains unclear, associations have been demonstrated with hemorrhagic disorders (due to anticoagulation, thrombolytic, or antiplatelet therapy), pregnancy, vascular malformations, neoplasms, and systemic diseases such as hypertension, diabetes, and hyperlipidemia [3,7,8]. These factors increase the risk of rupture of the spinal epidural venous plexus. In our case, the patient was on rivaroxaban for atrial fibrillation at the time of presentation.

The typical clinical presentation of SSEH involves an acute onset of neck or back pain followed by rapidly progressive neurological deficits. Although pain is often a predictor of SSEH, a recent review of cervical SSEH cases presenting with hemiparesis reported that 16 out of 51 patients did not experience neck pain [3]. Furthermore, sudden hemiparesis with neck pain can also occur in carotid artery dissection [9]. Therefore, pain alone is insufficient to distinguish SSEH from acute ischemic stroke.

In SSEH, tetraparesis or paraparesis is the most common neurological deficit. At the same time, hemiparesis is a rare presenting symptom [3,7,8], typically when the lesion involves the dorsolateral aspect of the cord [3], as in our patient. In the context of acute hemiparesis, misdiagnosis as ischemic stroke may lead to the administration of IV thrombolysis or antithrombotic therapy, which can worsen the patient’s condition [3]. Other clinical features that should raise suspicion for SSEH include the absence of cranial nerve deficits, ipsilateral Horner’s syndrome, Brown-Séquard syndrome, or Lhermitte’s sign [3]. These were absent in our patient. These were absent in our patient. Notably, although cranial nerve deficits are not expected in spinal cord pathology, mild dysarthria and ipsilateral facial sensory loss have been reported in SSEH [3], adding to the diagnostic challenge of distinguishing it from ischemic stroke.

Our patient exhibited some typical features of SSEH, including neck pain, paresis, absence of cranial nerve deficits, and anticoagulation use. However, his presentation with hemiparesis, without clear signs localizing the pathology to the spinal cord, created significant diagnostic uncertainty based on history and examination alone.

Neuroimaging is essential for confirming the diagnosis. MRI is the most sensitive modality for detecting SSEH [3,8], but it may not always be readily available. Given the narrow therapeutic window for acute ischemic stroke, cervical CT or CT angiography has been reported as an effective alternative for rapidly differentiating SSEH from stroke [3,10]. This was demonstrated in our case, where prompt diagnosis was achieved using CT angiography, thereby avoiding misdiagnosis. Significantly, when reviewing scans, physicians should not limit their focus to the brain parenchyma and vascular anatomy but should also carefully assess the cervical spine. Nonetheless, MRI remains valuable to distinguish intramedullary from extramedullary lesions, identify other pathologies such as cord infarction, hemorrhage, or malignancy, and guide surgical planning [3,8].

Management of SSEH includes surgical decompression and conservative therapy. Surgical evacuation via decompressive laminectomy is considered the standard of care for patients with severe or rapidly deteriorating neurological deficits [11]. Conservative management, on the other hand, has been reported to be effective in patients with mild symptoms and rapid or spontaneous neurological recovery [3,7,11]. Thrombolysis in SSEH has consistently been associated with poorer outcomes. In a review by Hu et al., among 13 patients presenting with hemiparesis who received IV thrombolysis, 10 experienced symptom aggravation, nine required surgery, and the remainder were treated conservatively [3].

## Conclusions

Although multiple case reports highlight SSEH as stroke mimics, few cases of SSEH were within the window for IV thrombolysis and were diagnosed with a CT angiogram only. Therefore, this case report serves to highlight, firstly, the need to recognize SSEH as a rare but potential stroke mimic, especially in cases presenting with acute neck pain without cranial nerve deficits, and secondly, the role of cervical CT angiograms in the early differentiation of acute ischemic stroke from SSEH.
